# Investigating the Polystyrene (PS) Biodegradation Potential of *Phanerochaete chrysosporium* Strain NA3: A Newly Isolated Soil Fungus

**DOI:** 10.3390/life15060869

**Published:** 2025-05-28

**Authors:** Muhammad Adnan Shereen, Sadia Mehmood Satti, Asim Abbasi, Naima Atiq, Qudsia Yousafi, Safia Ahmed, Kousar Parveen, Nazih Y. Rebouh

**Affiliations:** 1 Department of Microbiology, Kohsar University Murree, Murree 47150, Pakistan; 2 Alpha Genomics (Pvt), PWD Society, Islamabad 45320, Pakistan; 3 Department of Entomology, University of Agriculture, Faisalabad 38040, Pakistan; asimuaf95@gmail.com; 4 Department of Microbiology, Faculty of Biological Sciences, Quaid-i-Azam University, Islamabad 45320, Pakistan; 5 Department of Biosciences, COMSATS University Islamabad, Sahiwal Campus, Sahiwal 57000, Pakistan; 6 Department of Environmental Sciences, The Women University Multan, Multan 66000, Pakistan; 7 Department of Environmental Management, Institute of Environmental Engineering, RUDN University, 6 Miklukho-Maklaya St., Moscow 117198, Russia

**Keywords:** polystyrene, biodegradation, *Phanerochaete chrysosporium*, pretreatment

## Abstract

Biochemical monomer upcycling of plastic waste and its conversion into value-added products is deemed necessary, as it provides a greener and more sustainable solution to plastic waste management. In the current study, the polystyrene (PS) biodegradation potential of the fungus *Phanerochaete chrysosporium* NA3 was evaluated using various analytical techniques, such as Fourier transform infrared spectroscopy (FTIR), scanning electron microscopy (SEM), gel permeation chromatography (GPC), and high-performance liquid chromatography (HPLC). The biodegradation capacity of the fungal strain was further evaluated using a carbon dioxide (CO_2_) evolution test, which showed that the PS films treated with NA3 produced more CO_2_, indicating the strain’s ability to successfully utilize PS as a carbon source. The FTIR analysis of the PS films treated with NA3 showed modifications in the polymer chemical structure, including the formation of carbonyl and hydroxyl groups, which suggests the enzymatic dissociation of the polymer and the associated biodegradation mechanism. Pretreatments were found to be effective in modifying the polymer’s properties, making it more susceptible to microbial degradation, thus further accelerating the biodegradation process. The current study strongly advocates that *P. chrysosporium* (NA3) can be effectively used for the biochemical monomer recovery of PS waste and could be further utilized in the upcycling of plastic waste for its conversion into value-added products under the concept of circular economy.

## 1. Introduction

The synthetic organic polymers commonly known as plastics are low-cost, lightweight, robust, long-lasting, and corrosion-resistant materials with exceptional thermal and electrical insulating properties. Plastics and their associated materials have been extensively utilized in packaging, transportation, housewares, and buildings, easing humans in a number of ways [1,2]. However, the rapid accumulation of plastic waste into the environment is responsible for serious soil and groundwater pollution, while its incineration results in the emission of hazardous compounds into the environment [3,4]. Among the different sorts of plastics, polystyrene (PS) is the fifth most used material because of its valuable mechanical attributes and affordability [5,6]. The annual production of PS surpassed an amount of 32.6 million tons [7]. Extruded PS (XPS) and expanded PS (EPS), sometimes known as Styrofoam^®^, are commonly used in construction for packaging materials and insulation, and in disposable containers, including coffee cups and food trays. However, its widespread use has resulted in significant environmental concerns, primarily due to its non-biodegradability [8].

Polystyrene can persist in the environment for hundreds of years, and can accumulate in landfills, oceans, and other ecosystems. This accumulation is responsible for environmental contamination, often affecting humans and wildlife. The inability of PS to biodegrade is mainly due to its highly stable and inert nature, which also makes it resistant to microbial degradation. Therefore, PS waste remains in the environment for a very long time and causes severe environmental pollution [8]. Moreover, the linear carbon backbone with phenyl moieties gives PS its distinctive structure. This structural arrangement, combined with the lack of hydrolysable moieties, a common feature in all polyolefins, makes biodegradation extremely challenging [8]. Degrading PS has therefore emerged as a critical concern all around the globe. Currently, researchers are focusing on investigating the degradation of PS in activated sludge, compost, landfill sediment, and seawater.

The complex process of biodegradation of polymers is governed by a variety of factors, including both biotic and abiotic components of the environment as well as the properties of the polymers itself. Soil and composts have higher concentrations of microbes actively involved in the biodegradation process. However, these microbes are usually lacking in aquatic environments, including both freshwater and marine reserves [9]. Numerous researchers have identified a variety of microbes, including bacteria and fungi, that can break down both natural and synthetic polystyrene. Moreover, a limited proportion of bacteria and fungi can develop a biofilm on PS materials that can substantially alter the chemical composition of PS [8,10]. Similarly, worms also have the potential to biodegrade PS, which further supports the existence of a biological system that can efficiently work in the absence of light or additional heat [11]. The biodegradation of PS has also been carried out by certain soil-inhabiting bacteria, including *Microbacterium* sp. NA33 [12], *Paenibacillus urinalis* NA36 [13], *Bacillus* sp. NB6 [14], and *Pseudomonas aeruginosa* NB26 [15]. Therefore, in order to achieve sustainable and successful PS biodegradation, indigenous microbial fauna must be explored that possesses the potential of biotransformation of PS.

*Phanerochaete chrysosporium* is a soil-dwelling fungus that produces flat merged reproductive fruiting bodies, instead of a mushroom-like structure. The fungus belongs to the family *Phanerochaetaceae* [16]. Due to its effective ligninolytic abilities, rapid growth, and simple culture management, *P. chrysosporium* has evolved as a standard laboratory fungus with the potential to degrade PS [17]. The primary objective of the current investigation was to identify a possible PS-degrading fungal strain and assess its capacity for biodegradation of and bio-upcycling of PS.

In the current research, *Phanerochaete chrysosporium* was isolated from waste disposal soil and analyzed for its ability to degrade PS using a variety of analytical techniques, including Fourier transform infrared spectroscopy (FTIR), scanning electron microscopy (SEM), gel permeation chromatography (GPC), and high-performance liquid chromatography (HPLC).

The objectives of the current research were extracted from a previous study in which biodegradation potential of a white-rot fungus was assessed against polystyrene. However, our study is novel because it focuses on the colonization of the polystyrene film by soil fungi and uses denaturant gradient gel electrophoresis (DGGE) to identify the predominant colonizers. Additionally, we also investigated the microbial community structure and diversity in the soil adhered to buried polystyrene films using molecular biology techniques, including DGGE and scanning electron microscopy (SEM), under natural soil conditions. This combination of techniques allowed us to gain a better understanding of the real-world dynamics of polystyrene biodegradation in the soil environments.

## 2. Materials and Methods

### 2.1. Materials

Polystyrene used in the current study was obtained from FLUKA, Germany. The polymer resin has an average molecular weight (Mw) of 1 × 10^5^ Da. The growth medium, mineral saline medium (MSM), and different chemicals utilized in the study were purchased from the DIFCO labs (Detroit, MI, USA), the BDH laboratory chemical division (Pool Dorset, UK), FLUKA (Seelze, Germany), Merck, and Sigma Aldrich (Darmstadt, Germany). Moreover, Styrotech (Erdington, UK) provided the expanded polystyrene beads.

### 2.2. Preparation of Polystyrene Film

Chloroform was applied to dissolve the polystyrene (FLUKA, Seelze, Germany, Mol. wt. 100,000 Da) to make a 2% (*w*/*v*) solution. To get thin films of polystyrene (0.03–0.05 mm), the particles in the solution were homogeneously dispersed using a sonicator (Sonicator Heat Systema Ultrasonics INC cell disrupter model W225R, Qsonica, LLC, Newtown, CT, USA), poured into Petri plates, and left for one night. For use in subsequent biodegradation tests, the films were sliced into rectangular sections of about 6 cm × 2.5 cm.

### 2.3. Isolation of Fungal Strain with Polystyrene-Degrading Potential

The conventional soil burial and acclimatization procedure was followed to identify the microbe with the capability of biodegrading PS. The soil from the garbage disposal site at Quaid-e-Azam University, Islamabad, Pakistan, was collected and combined with manure in a 3:1 ratio. To boost microbial activity, a 2% solution of glucose was introduced into the soil. For 32 weeks, 500 g of extruded polystyrene films was submerged in the soil. After this, the films were taken out of the soil and thoroughly rinsed with sterilized distilled water to remove any remaining soil debris. The films were then sliced into squares and incubated on mineral salt media plates at 30 °C for seven days. The mineral salt media had no other carbon source and had the following content in g/L: KH_2_PO_4_ 0.2, Fe_2_(SO_4_)_3_; 6H_2_O 0.01, NaCl 1.0, ZnSO_4_; H_2_O 0.001, CaCl_2_; 2H_2_O 0.002, MgSO_4_; 7H_2_O 0.5, H_3_BO_3_ 0.005, CuSO_4_; 5H_2_O 0.001, NH_4_(SO_4_)_2_ 1.0, MnSO_4_; H_2_O 0.001; and K_2_HPO_4_ 1.0. For screening, each of the fungal cultures established on the plate was inoculated individually on potato dextrose agar plates. A polystyrene film was deployed as the major source of carbon in an Erlenmeyer flask comprising MSM and the identified fungal strains. For eight weeks, the flasks were maintained at 30 °C and 70 rpm in a shaking incubator. At the end of the investigation, the biodegradation capacity of the strains was determined by assessing any chemical alterations to the polymer’s composition using Fourier transform infrared spectroscopy (FTIR). The designated fungal strain with PS biodegradation capacity was retained for additional analysis on potato dextrose agar plates and slants.

### 2.4. Extraction of DNA from Fungal Mycelia

The fungal strain with the most promising capability for PS biodegradation was chosen, cultured on PDA broth for 72 h at 120 rpm and 30 °C, and harvested using a filtering process. After being washed twice to remove any leftover media, the filtered mycelia were air-dried using a vacuum pump before being ground into a powder. Using the previously described SDS lysis and isopropanol precipitation method of DNA extraction, the DNA of an isolated fungal strain was extracted [18]. The Nanodrop^TM^ 1000 and ND1000 3.1.0 software (Thermo Fisher Scientific Inc., Waltham, MA, USA) were utilized to quantify the extracted DNA, which was then kept at −20 °C for subsequent processing.

### 2.5. Amplification, Purification, and Sequencing of rDNA of the Isolated Fungal Strain

The ITS1-5.8S-ITS2 rDNA gene complex of the fungus was utilized for the molecular identification of the fungal isolate. Both ITS1 (5′ -TCCGTAGGTGAACCTGCGG-3′) and ITS4 (5′ -TCCTCCGCTTATTGATATGC-3′) were utilized as general fungal primers. The primary PCR reaction mixture contained 5 mL of template, 0.05 mM of dNTPs (Bioline Ltd., London, UK), 1xNH_4_ reaction buffer, and 0.2 mM each of the ITS1 and ITS2 primers. The final volume was adjusted to 50 mL using water that had been treated with diethylpyrocarbonate (DEPC). The PCR reaction was carried out under the following conditions: 94 °C for 1 min of denaturation, 56 °C for 1 min of annealing, and 72 °C for 1 min of extension. The PCR products were amplified, and then the agarose gel (1.5 percent *w*/*v*, Sigma Aldrich, Seelze, Germany) and DNA marker Hyper ladder IV (Bioline Ltd., London, UK) were used for gel electrophoresis. The PCR product was further purified using the QIAquick^®^ PCR Purification Kit (Qiagen Ltd., Crawley, UK) following the manufacturer’s instructions. Using a NanodropTM 1000 machine and ND-1000 3.1.0 software (Thermo Fisher Scientific Inc, Waltham, MA, USA), the DNA samples that had been purified were measured once more. The final mixture contained 2 pmol (0.2 mM) of primers (ITS1 and ITS4), 20 ng of purified PCR products, and an ultimate volume of around 10 mL that had been regulated with DEPC water. The University of Manchester Sequencing Facility received this final solution for Sanger sequencing. Using SeaView 4.6.1, a consensus sequence of the sequences was generated [19]. The identified fungus strain’s final consensus sequencing was uploaded to GenBank under the accession number FJ654431. The National Centre for Biotechnology Information (NCBI) database was explored using the Blastn algorithm to detect closely related species, which aided in identifying the fungus strain. A phylogenetic tree was created once the sequences for the closely related species were retrieved. A total of nine reference sequences and one outgroup were used. The MEGA 11 software’s ClustalW tool was used to align the sequences [20]. Using the neighbor-joining approach, the aligned sequences’ phylogenetic tree was created [21] and bootstrapped at 1000 repetitions [22].

### 2.6. Biodegradation Kinetics of Polystyrene Using Phanerochaete Chrysosporium Strain NA3

#### 2.6.1. Inoculum Preparation

The *Phanerochaete chrysosporium* spore suspension was prepared using sterile normal saline as the base solution. To enhance dispersal and prevent clumping, 20 mL of tween 20 (0.05% *v*/*v*) was added to tissue culture bottles containing the fungal strain cultured in triplicate. The bottles were thoroughly shaken to ensure proper mixing before transferring the solution into sterile collection tubes. To determine the spore concentration, the spore count per mL was calculated using Mod-Fuch’s Rosethal (0.2 mm Depth 1/116 mm^2^ WEBER England), which is a staining technique commonly used for fungal spore quantification.

To maintain the integrity of the inoculum during the experiments, a 0.5% concentration of streptomycin, an antibacterial agent, was applied. This was applied to minimize bacterial contamination that could potentially affect the stability of the fungal inoculum. The stability of the inoculum was assessed using viability assays and colony-forming unit (CFU) counts. Viability assays involve staining techniques or specific culture media that differentiate viable and non-viable cells. By employing such methods at different time points, the percentage of viable spores or cells in the inoculum could be determined. CFU counts, on the other hand, involved diluting the inoculum and plating it on suitable growth media. The resulting colonies were counted after an incubation period, providing an estimate of the viable fungal cells or spores. By comparing viability assays and CFU counts at various time points, we were able to evaluate the stability of the *Phanerochaete chrysosporium* inoculum throughout the experiments.

#### 2.6.2. Carbon Dioxide (CO_2_) Evolution Test (Sturm Test)

The mineralization of PS was assessed by monitoring oxygen consumption and carbon dioxide evolution as a result of carbon oxidization in the polymer. The isolated fungal strain NA3 utilized PS to generate carbon dioxide. The amount of carbon dioxide produced by the fungal strain was compared to the carbon dioxide produced by the abiotic control group under similar conditions. The biotic control group allowed the inoculation of fungus into the media without inoculation of the polymer film. About 300 mL of mineral salt medium with 250 mg of PS in the form of film fragments was added to the abiotic control and test jars. The spores of isolated fungus strain NA3 were introduced into the test jar at a concentration of 3 × 10^6^ spores per mL. The biotic and abiotic controls were likewise set up in parallel with continuous stirring under similar experimental conditions. Before passing through the culture bottles, the sterilized air was passed through the KOH (3 M) solution to remove CO_2_. The test was performed at 30 °C for 4 weeks. By employing barium chloride (1 M) to precipitate the soluble carbonates created in the absorption chamber, the CO_2_ generated in the test chamber was gravimetrically recorded and the dry mass was determined [23].

#### 2.6.3. Biodegradation of Polystyrene (PS) Films with Newly Isolated Fungal Strain NA3

The newly identified fungal strain NA3 was tested for its ability to biodegrade PS films. To prevent contamination on the surface of polymer films, the films were sterilized by immersing them in the 70% (*v*/*v*) ethanol solution and then rinsed with sterilized milliQ^®^ water. The inoculum was developed from scratch, as previously mentioned in Section 2.1. The films were added to a 250 mL Erlenmeyer flask that contained 100 mL MSM and 3 × 10^6^ fungal spores per mL of inoculum, and were then cultured for 16 weeks at 120 rpm at 30 °C. An abiotic control was also put up to detect whether there had been any abiotic deterioration. The experimental procedure was executed in triplicate.

#### 2.6.4. Evaluation of the Effect of Thermal and UV Pretreatment on the Biodegradation of Polystyrene (PS) Films

The PS films were exposed to UV light (230 V, 50 Hz, LF-106.L UVI tech Ltd., England) for 60 min to examine the impact of heat and UV treatment on the biodegradation of the films. The UV lamp utilized had a 254 nm wavelength, and the exposure distance was 3 cm. Similarly, the heat treatment was administered by placing the films in an oven at 80 °C for 1 h (LTE G215 Oven) [24,25]. The PS films pretreated with both UV and heat were added to a 250 mL Erlenmeyer flask containing 100 mL of MSM followed by inoculation of the fungal spores at a concentration of 3 × 10^6^ spores per mL. Then, the flask was incubated at 120 rpm and 30 °C for eight weeks. An abiotic control was also configured to ascertain any abiotic deterioration. Each experiment was run in triplicate.

#### 2.6.5. Evaluating Bioaugmentation of Fungal Strain NA3 to Enhance Soil Biodegradation of Polystyrene (PS)

Garden soil from Quaid-e-Azam University, Islamabad, Pakistan, was used to create soil microcosms. A homogenous soil sample was developed by taking the topsoil from three different locations, followed by blending them together and sieving through a 1 mm screen to get rid of the considerably larger soil particles. Using criteria outlined by the ISO, the physicochemical characteristics of the soil were evaluated [26,27]. To assess the biodegradation capacity of fungal isolates, particularly the effect of abiotic variables in the degradation process, the experimental procedure utilized both sterilized and unsterilized soils. The addition of distilled water brought the soil moisture level to 50%, which was maintained during the course of 8 months. The soil was autoclaved twice using a large tray at 121 °C for 30 min. The PS films (50 mg) were sterilized by exposing them to 70% ethanol for 5 min, followed by a water rinse. The films were placed in separate sterilized and unsterilized soil pots holding 50 g of soil, and then 10 mL of the fungus strain NA3 in MSM was added as an inoculant. The pots were incubated for 8 months at 30 °C. Negative controls were also set up, consisting of sterilized and unsterilized soil bearing plastic sheets inoculated with MSM without the identified fungal strain. After two, four, and eight months, samples of PS films were collected to assess biodegradation using the various analytical procedures mentioned below. All experimental treatments were set up in triplicate. Total viable count (CFU/mL) was used to calculate the total number of microorganisms in the soil [28].

To evaluate various fungal species adhering to the PS film buried in soil, denaturing gradient gel electrophoresis (DGGE) was utilized. For extraction of DNA from the surface of the PS, a DNA extraction kit (Fast Prep-24 MP^TM^ Fast DNA^®^Kit BIO 101 Systems Q-Biogene) was used. The ribosomal DNA sequences were amplified using DGGE-specific primers, with the forward primer (JB206c) having the sequence (5′CGCCCGCCGCGCGCGGCGGGCGGGGCGGGGGCACGGGG-GAAGTAAAAGTCGTAACAAGG3′), whereas the reverse primer GM2 had a sequence of (5′CTGCGTTCTTCTTCATCGAT3′) [29].

#### 2.6.6. Biodegradation of Polystyrene–Starch Blend Film by Fungal Strain NA3

A polystyrene and starch mixture was created by dissolving 5% starch (*w*/*w*) in PS using benzene as a solvent, with constant stirring at 80 °C. The solution was poured into a 25 cm-diameter Petri dish and then left for overnight air drying at room temperature. The final film was divided into 2 cm × 2 cm square fragments. By incubating the newly created blended film with the isolated fungal strain NA3 at 120 rpm and 30 °C for 8 weeks, it was determined how the addition of starch to the PS affected the biodegradability of the initial polymer.

### 2.7. Analysis of Biodegradation

By using the following analytical procedures, the biodegradation of polystyrene and expanded polystyrene was investigated.

#### 2.7.1. Fourier Transform Infrared Spectroscopy (FTIR)

The effect of biodegradation on the functional groups of the polymer was investigated using FT-IR spectroscopy in absorbance mode (Bio-Rad Merlin Excaliber) on films of polystyrene recovered from several trials. The absorbance of each sample was assessed twice in the 400–4000 cm^−1^ mid-IR spectral range. The abiotic control was also examined as a reference.

#### 2.7.2. Environmental Scanning Electron Microscopy (ESEM)

The fungus proliferation and surface alterations on the polystyrene sheet were examined using environmental scanning electron microscopy (ESEM) (FEI Quanta 200, Thermo Fisher Scientific Inc. Waltham, MA, USA). An LFD (large-field detector) and the low-vacuum 0.68 Torr mode were used for the analysis (Hitachi SU 1500, Tokyo, Japan). The ESEM was used to analyze the films that were recovered from all treatments. Samples were properly cleaned with distilled water and metallized with gold in order to improve electrical conductivity. Later samples were mounted on copper stubs.

#### 2.7.3. Chromatographic Techniques for the Analysis of Biodegradation

In the current study, the chromatographic techniques were used. A detailed description of each technique is mentioned below.

##### Gel Permeation Chromatography (GPC)

Gel permeation chromatography (Viscotek GPC max VE 2001GPC Solvent/Sample Module, RI Detector VE 3580, Malvern Panalytical Ltd., Malvern, UK) was used to assess the changes in molecular weight distribution. After being dissolved in tetrahydrofuran (THF) in a 2:1 (*w*/*v*) ratio, PS samples were filtered using a 0.45 µm PTFE filter. A filtered sample solution was introduced into a Waters Inc. gel permeation chromatography (GPC) device (Milford, MA, USA). For GPC experiments, a column set called PL 2MB500A was utilized. The mobile phase consisted of tetrahydrofuran (THF; Fisher Scientific). One mL per minute was chosen as the flow rate and 100 μL was used for the injection. THF 0.01% (*w*/*v*) was used to dissolve the PS samples before they were filtered using 0.45 µm syringe filters (Millex^®^-HV PVDF 13 mm). The temperature of the UV detector was set at 35 °C, and the column temperature was set to 30 °C. n-Dodecane was utilized as an identification marker. The absolute Mw and Mn of the PS when dissolved in tetrahydrofuran were determined using a universal calibration curve produced with polystyrene and the Mark–Houwink constants, K = 0.000174 L/g and α = 0.736.

##### High-Pressure Liquid Chromatography (HPLC)

HPLC was used to find the biodegradation products created during the biodegradation of polystyrene (Shimadzu, Nishinokyo Kuwabara-cho Nakagyo-ku, Kyoto, Japan). The SIL-20AC Prominence Autosampler, DGU 20A5 Prominence Degasser, CTO-10AS VP Shimadzu column oven, RF-10AXL Shimadzu Florescence Detector, and Spd-20A Prominence UV/VIS Detector made up the prominence liquid chromatography system. An LC solution tool was utilized to perform the analysis. For chromatographic analysis, a C18 column (Supleco INC LI Chrospher RP18 5 µL, 259 mm × 4.6 mm) was employed. Acetonitrile and water made up the mobile phase in a ratio of 7:3. The injection volume was 10 mL, and the flow rate was set at 1 mL/min. At a wavelength of UV 210 nm, the metabolites were observed. Before the analysis, the samples were first centrifuged and then filtered using 0.2 µm filter sheets. Two-phenyl ethanol, one-phenyl-1, two-ethanediol, phenylacetaldehyde, styrene oxide, and styrene were used as internal standards for the HPLC analysis of biodegradation products.

To quantify the samples, HPLC analyses were performed using calibration curves for each product. The calibration curves were prepared by analyzing known concentrations of the target compounds or standards. The standards used for HPLC analysis of biodegradation products were 2-phenyl ethanol, 1-phenyl-1, 2-ethanediol, phenylacetaldehyde, styrene oxide, and styrene. These standards were used to establish a relationship between the peak area or height observed in the chromatogram and the corresponding concentration of the compound. During the HPLC analysis, the samples were injected into the chromatographic system, and their peak areas or heights were recorded. By comparing the obtained peak areas or heights with the calibration curves, the concentrations of the target compounds in the samples were determined. The quantification was based on the linear relationship between the peak response and the concentration established by the calibration curves. This approach facilitated the accurate and reliable quantification of the target compounds in the samples analyzed using HPLC.

### 2.8. Statistical Analysis

The IBM SPSS software, (17.0.11), Tukey’s HSD (honestly significant difference) test, analysis of variance (one-way ANOVA), and regression line comparison were used to assess the data for statistical significance. A *p*-value of less than 0.05 was established as the threshold for significance.

## 3. Results

### 3.1. Isolation of Fungal Strain with Polystyrene-Degrading Potential

The isolated microorganisms belonged to the area of dumped soil. After eight months, the polystyrene-biodegrading microorganisms were isolated from the polystyrene. Fungal mycelia were found in the growth medium of MSM agar plates after 7 days of incubation. Initially, six fungal isolates were considered as candidate strains with PS biodegradation capacity. Further testing was carried out on the isolated fungal strains to see if they could biodegrade polystyrene in a liquid medium at 30 °C. Based on the results of FTIR spectroscopy, out of six, one fungal strain, i.e., *Phanerochaete chrysosporium* NA3, was selected for further biodegradation investigations due to its remarkable ability to degrade PS.

### 3.2. Molecular Identification of Fungal Strain

**Figure 1:**
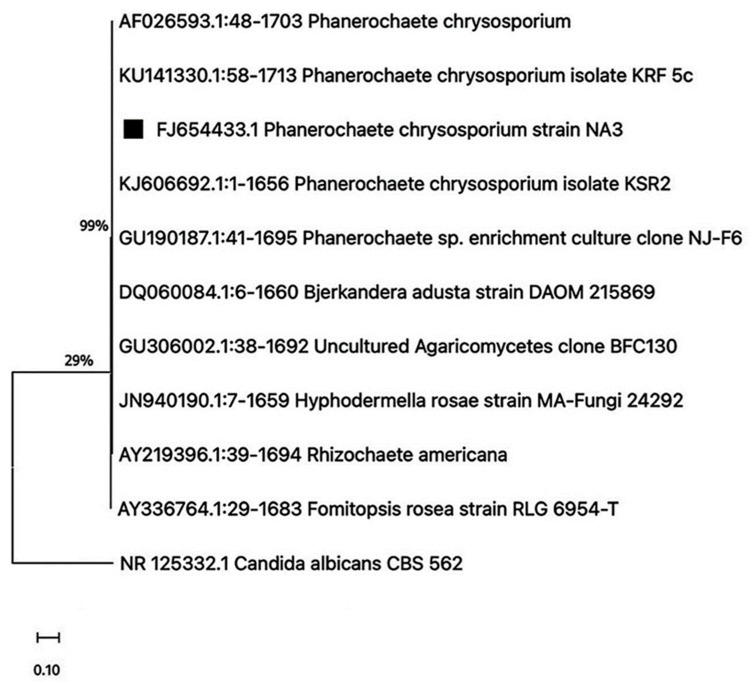
Evolutionary analyses of the isolated *Phanerochaete chrysosporium* fungus strain using the neighbor-joining method, bootstrapped at 1000 iterations. The bar represents 1% sequence divergence.

After successful extraction of DNA from the fungal mycelia, 18S rRNA PCR was performed, and for further confirmation of the fungal strains, a comparative study of 18S rRNA sequencing was performed. Sequences obtained through ITS sequencing were analyzed for sequence homology via BLAST 2.16.0 search in the NCBI nucleotide database. The acquired homologous sequences were then retrieved and aligned to construct a phylogenetic tree. The isolated strain was identified and given the title *Phanerochaete chrysosporium* strain NA3 (Figure 1) after showing maximal sequence homology to *Phanerochaete chrysosporium* (100%). The sequences were then submitted to NCBI GenBank, where accession numbers were generated (FJ654431).

### 3.3. Biodegradation Kinetics of Polystyrene Using Phanerochaete Chrysosporium Strain NA3

The concentration of *Phanerochaete chrysosporium* spores accounted for a concentration of 4.96 × 10^5^ spores per milliliter of solution, and *Phanerochaete chrysosporium* NA3 showed promising results in the biodegradation of polystyrene plastic.

#### 3.3.1. Carbon Dioxide (CO_2_) Evolution Test

**Figure 2:**
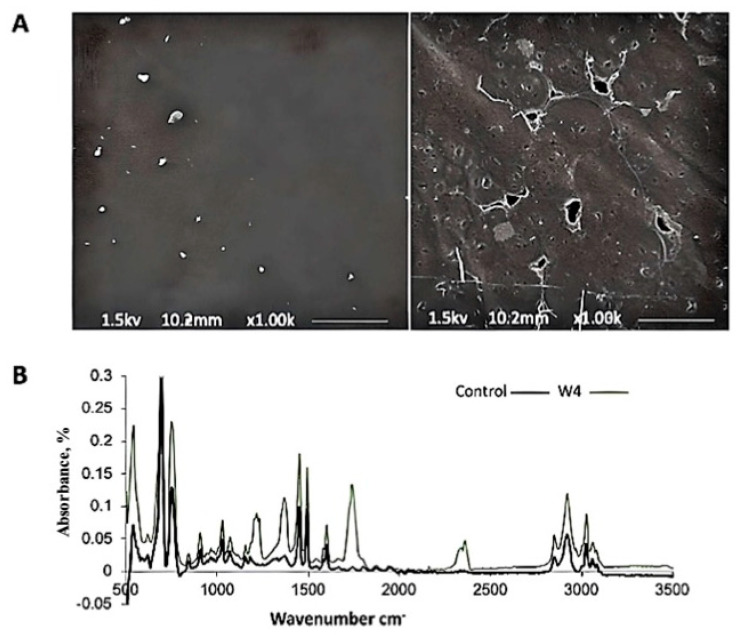
(**A**) Scanning electron micrograph (scale bar = 10.2 µm) after 4 weeks of incubation with fungal strain NA3 to evaluate the deterioration in PS film being utilized as the sole carbon source (**right**) compared to the abiotic control (**left**); (**B**) FTIR showing changes in functional groups in the abiotic control and films treated with fungal strain NA3 following 4 weeks of incubation at 30 °C.

The result confirmed the evolution of carbon dioxide during the biodegradation of polymers by *Phanerochaete chrysosporium* NA3. After 4 weeks of incubation, jars containing the PS polymer films treated with NA3 generated more CO_2_ compared to the biotic control. The experiment was carried out in triplicate. Table 1 shows the result of the carbon dioxide evolution test (Sturm test). Moreover, after the experiment, a considerable amount of fungal growth was observed on the PS sheet, which confirmed that the isolate has the capacity to use the film as a source of carbon. Environmental scanning electron microscopy (ESEM) further confirmed the significant fungal proliferation on the surface of the polystyrene sheet, as shown in Figure 2A: right side, as compared to the control with no fungal growth; left side: the capacity to effectively consume the polymer as a source of carbon, verified by the longer-term incubation of the polymer. The fungal strain with PS with a lack of any other carbon source showed effective growth and maximum carbon dioxide production.

Fourier transform infrared spectroscopy (FTIR) was further employed to evaluate the functional group modifications of polymer films incubated with the fungal strain. The spectra obtained from FTIR spectroscopy (Figure 2B) confirmed the changes in the chemical structure of the polymer. The result of the analysis confirmed that fungal strain NA3 had the capability to biodegrade the PS plastic.

#### 3.3.2. Biodegradation of Polystyrene (PS) Films with Newly Isolated Fungal Strain NA3 Under Shake Flask Conditions

**Figure 3:**
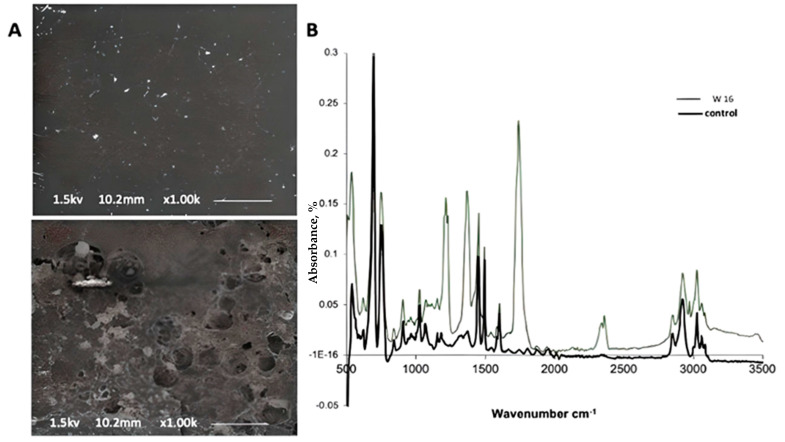
(**A**) Scanning electron micrograph (scale bar = 10.2 µm) after 4 weeks of incubation with fungal strain NA3 to evaluate the deterioration of PS film being utilized as the sole carbon source under shake flask conditions and at 30 °C (**below**) compared to the abiotic control (**above**); (**B**) FTIR spectra showing changes in the functional groups of the PS following biodegradation.

The results obtained from environmental scanning electron microscopy (ESEM) from the 16-week-old fungal-treated samples confirm the distinct adhesion and development of hyphae of the fungal isolate on the surface of the PS polymer (Figure 3A). Compared to the abiotic control sample, profusely grown fungus could be seen in the photograph of NA3 with the PS sample. Obvious cracks can easily be seen on the surface of the polymer surface compared to the abiotic control. The result confirmed the tendency of NA3 for biodegradation; moreover, the adhesion of the fungus mycelia to the polymer surface confirmed the compatibility of the microbe with the surface of the polymer.

The enzymatic dissociation of the polymer backbone and the chemical alterations in the polymer, which were apparent by FTIR spectroscopy, were the initial stages in the commencement of the biodegradation process. As a result of the depolymerization activity of *Phanerochaete chrysosporium* NA3, the FTIR spectra showed modifications to the functional groups of the polymer framework. The spectra demonstrated the emergence of new peaks and the removal of preexisting peaks in the test compared to the abiotic control, such as the appearance of new peaks at 1230 cm^−1^, 1377 cm^−1^, 1751 cm^−1^, 2364 cm^−1^, and 2368 cm^−1^ after 16 weeks of colonization with the fungal isolate. A similar increase in peak intensities was also noticed in the spectra (Figure 3B).

Gel permeation chromatography (GPC) analysis showed that the biodegradation of pure polystyrene by a fungal isolate was more feasible compared to that of the abiotic control, and changes in the molecular weight of the polymer were observed. The average molecular weight (Mw) of the control polystyrene film was 198,066 Da, and after 16 weeks of treatment by *Phanerochaete chrysosporium* NA, the Mw of the film of the fungal sample was 158,169 Da. The polymer’s average molecular weight (*Mn*) was measured as 107,553 Da throughout the control condition and 71,668 Da in the case of incubation with *Phanerochaete chrysosporium* NA3. After treatment with *Phanerochaete chrysosporium* NA3, the polydispersity index (Mw/Mn) was higher (2.248) relative to the untreated control (1.842) (Table 2).

**Figure 4:**
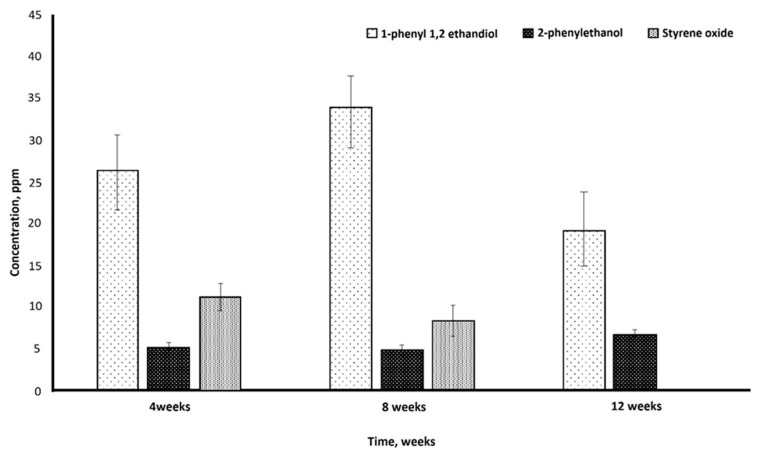
Detection of biodegradation products of PS upon incubation with *P. chrysosporium* strain NA3 under shake flask conditions and at 30 °C.

HPLC analysis confirmed the release of various degradation products from the biodegradation of the PS by the fungal isolate after 12 weeks of incubation. The highest concentration of 1-phenyl-1,2-ethanediol (34 ppm) was found after 8 weeks, while 2-phenyl ethanol was observed in small but consistent concentrations (2.5 ppm) in all the samples taken at the 4th, 8th, and 12th weeks of incubation. Styrene oxide was detected in samples taken in the 4th week (11 ppm) and 8th week (8.5 ppm) (Figure 4).

The results obtained from HPLC analysis represent active biodegradation of PS film by *P. chrysosporium* strain NA3.

### 3.4. Evaluation of the Effect of Thermal and UV Pretreatment on Biodegradation of Polystyrene (PS) Film

The PS samples were subjected to UV light for two hours and heat treatment at 60 °C for one hour to assess the effects of these pretreatments on the rate of biodegradation of the polystyrene film. Thereafter, the fungal isolate *Phanerochaete chrysosporium* NA3 was incubated under shake flask conditions. It is clear from the presentation of several analytical techniques that the polymer matrix was weakened during the process of thermal pretreatment of the PS films, which accelerated the rate of biodegradation after incubation with the fungal strain.

**Figure 5:**
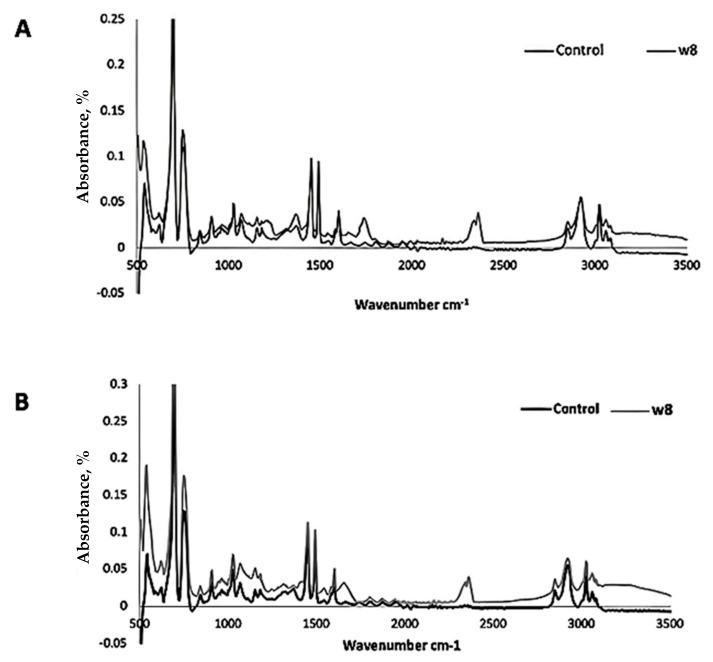
The FTIR spectra demonstrates the bond session of PS upon treatment with *Phanerochaete chrysosporium* NA3 after (**A**) UV treatment for 2 h and (**B**) heat treatment for one hour at 60 °C.

The FTIR spectra analysis showed that the UV-treated samples after incubation with the fungus showed diminished peak intensities compared to the abiotic control, including peaks at 756 cm^−1^, 1454 cm^−1^, 1476 cm^−1^, and 2420 cm^−1^. After 8 weeks of culture with the fungal isolate, the spectra indicated the formation of new peaks at 1759 cm^−1^, 2345 cm^−1^, and 2364 cm^−1^, as well as the removal of the preexisting peaks in the test. The spectra between 1000 cm^−1^ and 1270 cm^−1^ were modified, indicating the existence of hydroxy and ester groups (Figure 5A). FTIR spectra showed that the functional groups in the polymer structure were modified in the polymer films pretreated with heat and incubated with *Phanerochaete chrysosporium* NA3. After 8 weeks of incubation with the fungal isolate, additional peaks, i.e., at 1700 cm^−1^ and 2368 cm^−1^, in the test were observed compared to the abiotic control sample (Figure 5B).

Gel permeation chromatography (GPC) of heat-pretreated PS films revealed that heat treatment and incubation with *Phanerochaete chrysosporium* NA3 had degraded the films and that also was confirmed by an elevation in the polymer’s polydispersity index (3.426) as compared to the abiotic control (2.013) (Table 3). Contrary to the control, which had values of 232,142 and 73,191, respectively, the average molecular weight (Mw) and number (Mn) of the test were lower at 218,921 and 63,909.

An increase in the polydispersity index, measured by Gel Permeation Chromatography (GPC) was observed when *Phanerochaete chrysosporium* NA3 was irradiated with UV and incubated, as compared to the control (2.828) as shown in Table 3. In comparison to the control, which had values of 226,780 and 80,186, respectively, the average molecular weight (Mw) and number average molecular weight (Mn) dropped in the test case, (203,818 and 60,118, respectively).

### 3.5. Biodegradation of PS in Soil; Evaluating Bio-Augmentation of Phanerochaete Chrysosporium Strain NA3

For bioaugmentation analysis, PS films were buried underground and simultaneously subjected to abiotic control in sterile and non-sterile conditions, as well as inoculation by the fungus. The results of the experiment show that the abiotic control sample under sterilized conditions showed the least amount of biodegradation, followed by the control under non-sterile conditions. The results show that our fungal strain performed better in isolation, and that the bioaugmentation of our fungal strain under sterilized conditions showed greater biodegradation compared to unsterilized conditions.

**Figure 6:**
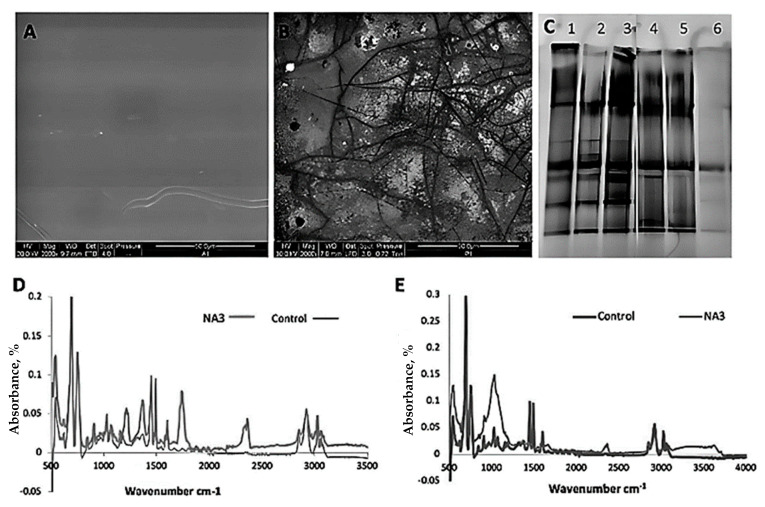
Micrographs (scale bar = 10.2 µm) showing the deterioration of PS film buried in soil upon bioaugmentation with *Phanerochaete chrysosporium* strain NA3: (**A**) unsterilized inoculated, (**B**) sterilized inoculated, (**C**) denaturing gradient gel electrophoresis showing dominant fungal colonizers attached to the polymer film (lanes 1–5: NA3, along with other native fungal colonizers of the unsterilized soil; lane 6: only NA3 in sterilized soil), (**D**) FTIR spectra showing changes in functional groups in the polymer film buried in unsterilized soil inoculated with fungal strain NA3, and (**E**) FTIR spectra showing changes in functional groups in the polymer film buried in sterilized soil inoculated with fungal strain NA3.

The SEM examination for the polymer film, when buried in sterile or non-sterile soil and incubated with a fungal isolate, showed surface deterioration of the polymer film in comparison to films buried in sterile or non-sterile soil that had not been inoculated with the fungal strain. The surface of the polymer film showed clear cracks, while only the sterile soil without fungal inoculation showed no impact on the polymer surface and appeared intact. After injecting the soil with fungal isolate, despite the soil type, clearer and broader cracks were found on surface of the film. The surface of the unsterilized soil with inoculated fungus was found to be rougher than other conditions, and significantly larger gaps were also visible (Figure 6A,B).

Denaturant gradient gel electrophoresis (DGGE) analysis for the fungal community adhering to and growing over the PS film surface was used to evaluate the guanine and cytosine (GC) content and heat stability. The DGGE gel depicted numerous bands in the case of the samples from unsterile soil, showing there were more fungal species attached to the PS, while in the case of the sterilized soil, one band was found showing the inoculated fungal strain NA3 (Figure 6C). The denaturant gradient gel electrophoresis analysis of the soil adhering to buried polystyrene films showed the presence of analogous dominant bands. Colonization of the polymer by fungal isolates can be seen in the scanning electron micrographs of the buried films.

The Fourier transform infrared spectroscopy (FTIR) analysis of the PS film showed a rise in absorbance intensities in the peaks at different places, representing the fungal isolate’s ability for biodegradation (Figure 6D,E). On the other hand, in the control sample, the films buried in unsterilized soil and injected with the fungal strain NA3 showed the formation of new peaks at 1037 cm^−1^, 2376 cm^−1^, and 3640 cm^−1^. The films that were buried in sterilized soil and inoculated with the fungal strain NA3 showed the formation of new peaks at 1230 cm^−1^, 1373 cm^−1^, 1743 cm^−1^, and 2364 cm^−1^.

To assess any potential reduction in the polymer’s molecular mass, gel permeation chromatography was performed. Table 4 represents the molecular weight and polydispersity of the PS samples dumped in both unsterilized and sterilized soil inoculated with the fungal isolates, showing a decrease in value compared to the controls. In unsterile soil samples, the weight average molecular weight (Mw) decreased more in the *Phanerochaete chrysosporium* NA3-inoculated soil than in the control, with relatively specific patterns observed in sterile soil.

### 3.6. Biodegradation of Polystyrene Starch Blend

**Figure 7:**
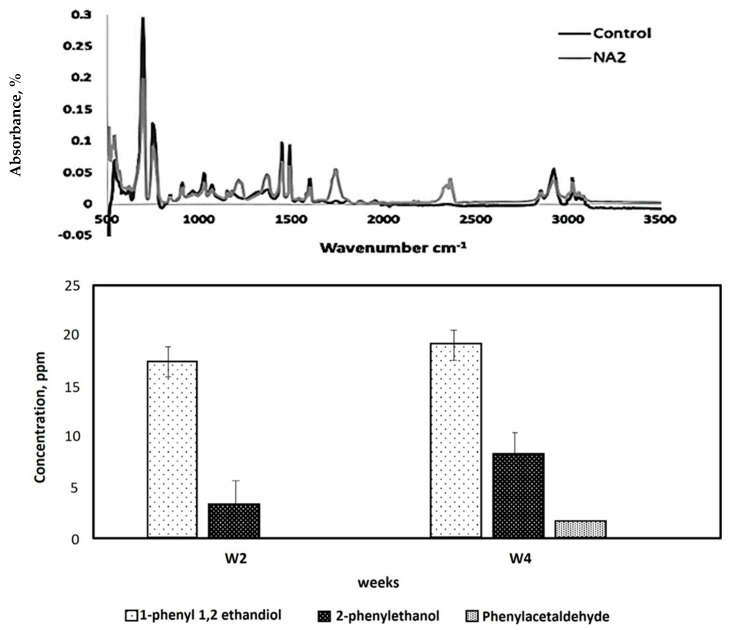
FTIR spectra showing changes in the functional group of PS upon incubation with isolated fungal strain NA3 compared to the abiotic control under shake flask conditions and at 30 °C (**above**). Detection of released biodegradation products of PS upon incubation with *P. chrysosporium* (**below**).

The purpose of studying the biodegradation of PS–starch mixtures is to evaluate how the addition of a natural, biodegradable polymer like starch can enhance the overall biodegradability of polystyrene (PS), which is otherwise highly resistant to microbial degradation. Starch serves as a co-substrate that can stimulate microbial activity by providing an easily metabolizable carbon source, thereby potentially accelerating the breakdown of PS [25]. Biodegradation of a blend of PS and starch, isolated by fungal strain NA3, was performed. Fourier transform infrared spectroscopy (FTIR) analysis of the PS–starch blend films showed the appearance of new peaks at 1223 cm^−1^, 1381 cm^−1^, 1751 cm^−1^, and 2364 cm^−1^, representing the C=O stretch of carbonyl groups such as ketones, aldehydes, and a carboxylic acid, and 1223 cm^−1^ to 1381 cm^−1^ represents the C-O vibrations of ester groups, including the emergence of nascent peaks at 2349 cm^−1^ and 2364 cm^−1^ (Figure 7, above).

HPLC analysis of the biodegradation products confirmed the presence of 1-phenyl-1,2-ethanediol and 2-phenylethanol in all samples inoculated with fungal strain NA3 and PS–starch blend films compared to the abiotic control. After 4 weeks of inoculation with *Phanerochaete chrysosporium* strain NA3, a maximum concentration of 1-phenyl-1,2-ethanediol (17.7 ppm) and 2-phenyl ethanol (4.5 ppm) was found in the sample (Figure 7, below). Meanwhile, the phenylacetaldehyde concentration was found only in the fourth week of incubation of the test flask.

## 4. Discussion

### 4.1. Isolation, Identification, and Phylogenetic Analysis of PS-Degrading Fungi

The current research was carried out to identify potential PS-degrading microorganisms and assess the strains’ capacity for biodegradation. Further identification of the isolated fungus was accomplished by a comparative study of 18S rRNA sequences, and the strain was identified as *P. chrysosporium*. Biodegradation of PS by microorganisms and larvae of different organisms has previously been reported in the literature, such as *Exiguobacterium* sp. strain YT2 [30], *Bacillus* spp., *Pseudomonas* spp. [31], *Enterobacter* spp [32], *Klebsiella* sp., *Micrococcus* sp., *Pseudomonas* sp. [33], *Pseudomonas putida* [34], *Penicillium variable* [35], *Citrobacter* sp., *Kosakonia* sp., [36], *Exiguobacterium* sp. DR11 [37], yellow mealworms (larvae of *Tenebriomo liter* Linnaeus), [38,39], *Tribolium castaneum* (red flour beetle) imago [40], *Tenebrio molitor* larvae [41], larvae of *Zophobasatratus* [42], and *Galleria mellonella* [43]. Although certain microorganisms have previously been identified as having the ability to biodegrade PS, the variety of microbes in this area has not been extensively investigated, and currently, there is a need to seek potential microbial strains to use in plastic waste management processes using upcycling technology. In the context of ever-growing plastic trash, this waste may be efficiently controlled via bio-upcycling of the plastic waste, and for this purpose, enzymatic depolymerization would be optimal, with improved carbon efficiency. This has made it necessary to look for suitable microbial strains and enzymes that might effectively degrade plastics.

When it comes to the biodegradation of polystyrene (PS), fungi have a number of advantages over bacteria. Fungal species can grow and multiply in a variety of ecological settings, such as dry soils and low nitrogen levels, where bacteria might not be able to survive. This gives fungi an advantage for degrading plastics in natural settings. Additionally, fungi usually grow slowly and release enzymes for extended periods of time, supporting ongoing of plastic materials [44,45]. Moreover, fungi can adhere to the plastic surface by their capacity to create stable mycelial networks, which keeps them in close proximity during the biodegradation process. Furthermore, fungi naturally colonize inert surfaces like plastics, which increases their enzymatic activity and adhesion. Fungal species can easily spread and start degradation in a variety of settings due to their capacity to generate spores. For large-scale or in situ applications, fungal decomposition could be more economical since it is less reliant on an external nutrition supply [44,45,46]. Complex and resistant polymers like PS can be broken down by a variety of extracellular oxidative enzymes produced by fungi, especially filamentous species. These enzymes include laccases, peroxidases, and monooxygenases. Their hyphal development increases surface contact with the polymer by enabling deeper penetration and colonization of solid substrates. Furthermore, the production of organic acids and reactive oxygen species, which aid in polymer depolymerization, is frequently favored by fungal metabolic pathways. Moreover, they can function as powerful single-organism degraders, in contrast to bacteria, which might be more dependent on surface-level degradation or need consortia for effectiveness [8,9,10,44,45,46].

Considering this, every report of a new microbial strain with the ability to decompose polymers like polystyrene is highly encouraging and offers significant optimism for a long-term approach to the conundrum of plastic waste through biochemical monomerization and further upcycling.

### 4.2. Assessing Biodegradation and Mineralization of Polystyrene (PS) Through Carbon Dioxide (CO_2_) Evolution Test

Carbon dioxide (CO_2_) evolve during the biodegradation of polymers by microbes due to several underlying processes and metabolic pathways. Microbes break down the polymer chains through enzymatic activities to access the carbon and energy sources present in the polymer. During this process, microbes utilize the polymer as a carbon and energy substrate for their growth and reproduction. The breakdown of polymer chains releases carbon in the form of carbon-containing compounds. Ultimately, these carbon compounds are metabolized by microbes, leading to the production of CO_2_ as a byproduct through the process of microbial respiration. To ascertain how the designated fungal strains degraded polystyrene, the carbon dioxide evolution test (Sturm test) was carried out (Table 1). The purpose of the Sturm test was to report or evaluate the complete mineralization and assimilation of PS, thus directly determining the biodegradation potential of the microbial strain [47,48]. After 4 weeks of culture at room temperature (~30 °C), it was revealed that the test jar, comprising the polymer films treated with NA3, generated more CO_2_ than the biotic control.

A close examination of the polymer film after the experiment revealed considerable fungal growth on the PS sheet surface and its capacity to use the film as a carbon source. Additionally, less CO_2_ was produced when a mineral salt medium with less carbon was used, demonstrating the reliance on PS as a carbon source. After 4 weeks of colonization, environmental scanning electron microscopy (ESEM) revealed significant fungal proliferation on the surface of the polystyrene sheet (Figure 2A). The fungal isolates were able to stick to and thrive on the PS surface, using the film as a reservoir of carbon. The capacity of the fungus to effectively consume the polymer was verified by longer-term incubation of the PS polymer with the fungal strain with a lack of any other carbon source. This also promoted biofilm formation on the polymer surface and adherence to the polymer [49,50]. In the biodegradation studies conducted using mineral salt media, where carbon was limited, only a small quantity of CO_2_ was generated [51]. Fourier transform infrared spectroscopy (FTIR) was further employed to evaluate the functional group modifications of polymer films incubated with the fungal strain. FTIR spectroscopy details enabled the evaluation of the changes in the chemical structure of the polymer. Microbes usually carry out the oxidative cleavage of the bonds in the polymer structure utilizing their enzymatic machinery. Therefore, due to oxidation, there are chances for the formation of carbonyl (>C=O) as well as hydroxyl (–OH) groups in the polymer structure [52], which is apparent in the spectra presented in Figure 2B. The Fourier transform infrared spectroscopy (FTIR) assessment of films retrieved after incubation with NA3 showed clearly that the test film had a series of additional peaks that had not been present in the control film. A new peak at 1230 cm^−1^ reflected the stretching of the ester group’s C-O atoms, while peaks at 1354 cm^−1^ revealed the stretching of an aromatic ring’s C=C linkages, while the peak at 1747 cm^−1^ revealed the stretching of an ester link, and the peaks appearing at 2372 cm^−1^ and 2376 cm^−1^ were likely due to atmospheric CO_2_ interference rather than functional group vibrations associated with polystyrene biodegradation (Figure 2B).

With this deeper insight into the chemical changes occurring during the biodegradation process, it was observed that microbes usually carried out oxidative cleavage of the bonds in the polymer structure utilizing their enzymatic machinery, leading to the formation of carbonyl (>C=O) and hydroxyl (–OH) groups in the polymer structure. The FTIR spectra presented in Figure 2B show the emergence of new peaks in the test film incubated with NA3 that were not present in the control film. These spectral changes indicate the enzymatic dissociation of the polymer backbone and the chemical alterations in the polymer. The oxidation of the polymer backbone led to the formation of carbonyl and hydroxyl groups in the polymer structure, which could further react with the fungal enzymes and accelerate the biodegradation process. The emergence of new peaks in the FTIR spectra also suggests the formation of new chemical bonds between the polymer and the fungal enzymes, further supporting the biodegradation mechanism. In conclusion, the results from the CO_2_ evolution test and the FTIR analysis suggest that the newly isolated fungal strain NA3 can biodegrade PS through oxidative cleavage of the polymer backbone and the formation of carbonyl and hydroxyl groups. These chemical alterations facilitate the enzymatic dissociation of the polymer backbone, leading to the biodegradation of the PS by the fungal strain.

### 4.3. Assessing the Biodegradation Potential of Polystyrene (PS) Films Using a Novel Fungal Strain NA3 Under Shake Flask Conditions

To assess the biodegradability of the PS with the isolated fungal strain, the polystyrene film was incubated with newly isolated *Phanerochaete chrysosporium* strain NA3 at 30 °C under shake flask conditions. Using environmental scanning electron microscopy (ESEM), the distinct adhesion and development of hyphae of the fungal isolate on the polymer surface were determined. After 16 weeks of incubation, the ESEM micrographs revealed a fungus growing profusely on the polystyrene film, in contrast to the abiotic control (Figure 3A). Comparing the polymer’s surface to the abiotic control revealed obvious cracks, which suggests the isolated fungal strain’s propensity for biodegradation. The adhesion of the fungus mycelia to the polymer surface demonstrates the compatibility of the microbe and the surface of the polymer. The enzymatic dissociation of the polymer backbone and the chemical alterations of the polymer, which were apparent with FTIR spectroscopy, are the initial stages in the commencement of the biodegradation process. As a result of the depolymerization activity of *Phanerochaete chrysosporium* NA3, the FTIR spectra showed modifications to the functional groups of the polymer framework. The spectra demonstrated the emergence of new peaks and the removal of the preexisting peaks in the test compared to the abiotic control, such as the appearance of new peaks at 1230 cm^−1^, 1377 cm^−1^, 1751 cm^−1^, 2364 cm^−1^, and 2368 cm^−1^ after 16 weeks of colonization with the fungal isolate. A similar increase in peak intensities was also noticed in the spectra (Figure 3B). The appearance of a new peak at 1751 cm^−1^ denoted the C=O functional group, which is an indication of an improvement in carbonyl operability and demonstrates the deterioration of the polymer’s amorphous portion and the appropriate growth of its crystalline region. The peak at 1230 cm^−1^ exhibited the ester group’s C-O stretching vibration, while the peak at 1377 cm^−1^ showed the CH_2_ group’s symmetric vibration. Furthermore, the appearance of the peak at 1751 cm^−1^ representing carboxylic functionality indicated hydrolysis of the polymer chains, resulting in the formation of carboxylic acids, as indicated by the peak at 1230 cm^−1^ [50,53].

Using gel permeation chromatography (GPC), it was feasible to evaluate the biodegradation of pure polystyrene by a fungal isolate to an abiotic control and observe how the polymer’s molecular weight changed. The control polystyrene film’s weight average molecular weight (Mw) was 198,066 Da, but the Mw of the film after 16 weeks of fungal treatment by *Phanerochaete chrysosporium* NA3 was 158,169 Da. The polymer’s average molecular weight (*Mn*) was measured as 107,553 Da throughout the control condition and 71,668 Da in the case of incubation with *Phanerochaete chrysosporium* NA3. After treatment with *Phanerochaete chrysosporium* NA3, the polydispersity index (Mw/Mn) was higher (2.248) relative to the untreated control (1.842) (Table 2). The reduction in *Mn* represents the bulk degradation happening in the polymer structure, which has been previously reported in the biodegradation of other polymers as well [54,55,56]. The drop in the value of Mw is linked to the polymer’s chain session and represents fragmentation; similarly, the rise in polydispersity index (PDI) also reflects the polymer’s chain distribution and the emergence of its shorter chains. The exact description of polydispersity is the quantity and distribution of chain lengths inside a polymer structure. Low polydispersity indicates a limited range of polymer chain lengths, whereas high polydispersity indicates a wide variety of chain lengths.

Upon biodegradation of the PS with a fungal isolate for 12 weeks, the release of various degradation products was detected using HPLC. The highest concentration of 1-phenyl-1,2-ethanediol (34 ppm) was found after 8 weeks, while 2-phenyl ethanol was observed in small but consistent concentrations (2.5 ppm) in all the samples taken at the 4th, 8th, and 12th weeks of incubation. Styrene oxide was detected in samples taken at the 4th week (11 ppm) and 8th week (8.5 ppm) (Figure 4).

The results obtained from HPLC analysis represent active biodegradation of PS film by *P. chrysosporium* strain NA3. The inferences obtained from HPLC in correlation with the FTIR observations demonstrate that PS biodegradation takes place as oxo-biodegradation [57], a relatively slow process [58]. The oxo-degradation process resulted in the formation of low-molecular-weight products such as alcohols, carboxylic acid, aldehydes, and ketones, which might have been quickly assimilated in the cells and thus not been detected in HPLC [59,60].

### 4.4. Evaluation of the Effect of Thermal and UV Pretreatment on Biodegradation of Polystyrene (PS) Film

It is very well reported that pretreatment strategies like UV irradiation and heat treatment have the potential to weaken and modify the polymer structure to promote the biodegradation of the polymer. Such pretreatments promote cross-linking among the polymer chains, thus enhancing the bond cleavage and resulting in a reduction in the molecular weight distribution; furthermore, polar functional groups arise in the polymer matrix, thus promoting the hydrophilicity of the polymer surface. Under aerobic conditions, protons are abstracted from a polystyrene chain, and oxygen is added due to exposure to UV radiation, leading to β-scission [61]. Similarly, thermal or radiation treatments on polymers reduce the polymeric chain size and form oxidized groups such as carboxyl, carbonyl, and hydroxyl, which are more easily degraded by microorganisms [62]. Oxidized groups modulate microbial attachment by increasing surface hydrophilicity as well [62]. Therefore, polymer degradation is expected to be enhanced if a more oxidized surface is used as a substrate [63]. These treatments are known to modify properties such as crystallinity level and morphological changes of the original polymer and facilitate polymer biodegradation [64].

The FTIR spectra were used to evaluate the breakage of bonds and changes in the chemical composition through the functional groups’ displacement of the polymer structure [65] as a result of the depolymerization activity of the *Phanerochaete chrysosporium* NA3 after heat treatment of the polymer. The UV-treated samples after incubation with fungal isolates showed diminished peak intensities compared to the abiotic control, including peaks at 756 cm^−1^, 1454 cm^−1^, and 1476 cm^−1^. After 8 weeks of culturing with the fungal isolate, the spectra indicated the formation of new peaks at 1759 cm^−1^, 2345 cm^−1^, and 2364 cm^−1^, as well as the removal of the preexisting peaks in the test. The spectra between 1000 cm^−1^ and 1270 cm^−1^ were modified, indicating the existence of hydroxy and ester groups (Figure 5A). Similar modifications in the functional groups of the polymer structure were seen in the FTIR spectra of the polymer films pretreated with heat and incubated with *Phanerochaete chrysosporium* NA3. After 8 weeks of incubation with the fungal isolate, the spectra demonstrated the emergence of additional peaks in the test compared to the abiotic control, such as a new peak at 1700 cm^−1^. The appearance of a new peak at 1751 cm^−1^ was assigned to the C=O stretching. The vibration of a ketone group showed that thermal pretreatment promoted the creation of ester- and keto-carbonyl groups in the polymer structure, which further promoted the microbial degradation of the polymer (Figure 5B).

After UV irradiation and incubation with *Phanerochaete chrysosporium* NA3, the polydispersity index of the polymer increased, as measured by gel permeation chromatography (GPC), compared to the control (2.828) (Table 3). In comparison to the control, which had values of 226,780 and 80,186, respectively, the average molecular weight (Mw) and number average molecular weight (Mn) dropped in the test case, (203,818 and 60,118, respectively). This demonstrates that the polymer matrix had been compromised and that the polymer chains had been decomposed into shorter chains of variable lengths, exposing the terminal functional groups in the polymer backbone that could be instantly manipulated by microbes and accelerating the rate of biodegradation.

The UV-treated PS films showed a decrease in Mw and Mn, an increase in the polymer’s polydispersity index, and a modification in the functional groups of the polymer structure, leading to faster biodegradation after incubation with the fungal strain. The FTIR spectra showed the formation of new peaks and the removal of preexisting peaks in the test compared to the abiotic control, indicating the creation of hydroxy and ester groups in the polymer structure, which are more susceptible to microbial degradation.

The use of pretreatments such as UV irradiation and heat treatment has been found to promote the biodegradation of polystyrene by modifying the polymer structure, resulting in the formation of oxidized groups such as carboxyl, carbonyl, and hydroxyl, which are more easily degraded by microorganisms. These treatments also increase the surface hydrophilicity of the polymer, which enhances microbial attachment, leading to faster biodegradation.

The findings provide important insights into the mechanisms underlying the biodegradation of polystyrene, particularly in the context of fungal degradation. The study demonstrates that the isolated fungal strain *Phanerochaete chrysosporium* NA3 is capable of biodegrading polystyrene, with clear evidence of enzymatic dissociation of the polymer backbone and the emergence of new peaks in the FTIR spectra indicating modifications to the functional groups of the polymer structure. The study also shows that pretreatment strategies like UV irradiation and heat treatment can weaken and modify the polymer structure, promoting the biodegradation of the polymer. These results also suggest potential strategies for enhancing the biodegradation of polystyrene, such as using pretreatments to modify the polymer’s properties to make it more susceptible to microbial degradation. Additionally, the study highlights the potential of using fungal strains like *Phanerochaete chrysosporium* NA3 for the biodegradation of polystyrene. The study’s results could inform the development of more efficient and sustainable biodegradation methods for polystyrene, which is a widely used polymer and a major environmental pollutant.

### 4.5. Biodegradation of PS in Soil; Evaluating Bio-Augmentation of Phanerochaete Chrysosporium Strain NA3

Polystyrene is a very slow-degrading polymer due to the presence of phenyl side groups in the structure [66]. To comprehend and assess the true potential of this strain in a real soil environment, it is essential to improve the process of biodegradation. This might be done by introducing the concept of bioaugmentation. In this approach, the PS films were buried underground and simultaneously subjected to abiotic control in sterile and non-sterile instances, as well as to inoculation by the fungus. After the trial, several analyses were applied to assess the fungus’s actual capacity for biodegradation. The abiotic control under sterilized circumstances showed the least amount of biodegradation, followed by the control under non-sterile conditions. This may be explained by the fact that negligible biodegradation was observed under sterilized conditions due to the paucity of microbial activity in the abiotic control, which also emphasizes the low contribution of abiotic components to biodegradation. The results show that our fungal strain performed better in isolation and may not have formed a synergistic relationship with the soil population, as the bioaugmentation of our fungal strain under sterilized conditions resulted in greater biodegradation than the unsterilized conditions.

In both cases, when the polymer film was buried in sterile or non-sterile soil and incubated with a fungal isolate, the SEM examination showed how rough the surface of the polymer film was in comparison to films buried in sterile or non-sterile soil that had not been inoculated with the fungal strain. When the film was buried in all types of soil except sterile soil without fungal inoculation, cracks and burrows were seen in the film. When soil was injected with fungal isolate, whether it was sterile or not, clearer and broader cracks were seen. The surface of the polymer film buried in the unsterilized soil, where our fungus was inoculated, appeared to be rougher than those under other conditions, and significantly larger gaps were also detected (Figure 6A,B).

PS films were placed in soil under controlled setups with the fungal isolates, and the Fourier transform infrared spectroscopy (FTIR) analysis revealed a rise in absorbance intensities in the peaks at different places, representing the fungal isolate’s ability for biodegradation (Figure 6D,E). In contrast to the control, the films buried in unsterilized soil and injected with the fungal strain NA3 showed the formation of new peaks at 1037 cm^−1^, 2376 cm^−1^, and 3640 cm^−1^. Comparable to the control, the films that were buried in sterilized soil and inoculated with the fungal strain NA3 showed the formation of new peaks at 1230 cm^−1^, 1373 cm^−1^, and 1743 cm^−1^. It is evident that when compared to the other treatments, the films that were planted in soil that had been sterilized and inoculated with a fungal isolate exhibited more biodegradation. The emergence of two additional peaks at 1743 cm^−1^ signified the stretching of the C=O bonds of a ketone group. The peak at 1373 cm^−1^ is referred to as the C-H bond bending symmetric vibration of the CH_2_ group. This indicated the differences between the fungal strain-treated polymer and the control in terms of basic chemical structure. The fungal community adhering to and growing over the PS film surface was evaluated by denaturant gradient gel electrophoresis (DGGE) separating the genomes based on their GC content and heat stability. This technique is used to identify microbial communities present in environmental samples. The DGGE gel depicted numerous bands in the case of the samples from unsterile soil showing that there were more fungal species attached to the PS, while in the case of the sterilized soil, one band was found showing the inoculated fungal strain NA3 (Figure 6C). The denaturant gradient gel electrophoresis analysis of the soil adhering to buried polystyrene films revealed the presence of analogous dominant bands, suggesting that specific soil fungi were the predominant colonizers of the polymer. Additionally, the scanning electron micrographs of the buried films illustrated the colonization of the polymer by these fungi.

Denaturant gradient gel electrophoresis (DGGE), a method for identifying microbial communities in environmental samples, was employed to assess the fungal population adhering to and expanding over the PS film surface. In samples of non-sterile soil, the DGGE gel displayed multiple bands, indicating a wide variety of fungus species adhering to the PS. The injected fungal strain NA3 was only visible in one band in soil samples that had undergone sterilization. According to the DGGE examination of soil that was adherent to buried PS films, a particular type of soil fungus was the polymer’s main colonizer.

The value of the DGGE analysis is found in its capacity to recognize and evaluate the microbial communities that were present in soil samples with and without the injected fungal strain. This comparison can be used to determine whether fungal strain NA3 influences biodiversity during PS biodegradation and how it interacts with the already-existing microbial community. A fuller understanding of the microbial dynamics and their impact on PS degradation would be made possible by additional explanation and interpretation of the DGGE results.

Gel permeation chromatography was performed to assess any potential reduction in the polymer’s molecular mass. According to Table 4, the molecular weight and polydispersity of the PS samples dumped in both unsterilized and sterilized soil that had been inoculated with the fungus decreased compared to the controls. In unsterile soil samples, the weight average molecular weight (Mw) decreased more in the *Phanerochaete chrysosporium* NA3-inoculated soil than in the control, with relatively specific patterns observed in sterile soil. The decrease in the Mn suggests that the polymer chains had been chopped by the microbial attack and that smaller fragments were produced [50,67].

### 4.6. Biodegradation of Polystyrene Starch Blend

The biodegradation of the PS film mixed with starch (5% *w*/*w*) was assessed after being inoculated with the identified fungal strain NA3 for 8 weeks under shake flask conditions. Blending synthetic polymers with substances that have hydrolysable groups might accelerate the biodegradation of refractory polymers, particularly PS. The hydrolysable component acts as the precursor for the start of the biodegradation; the structure of the polymer matrix is modified and the surface area is enhanced, which promotes enzymatic attack by the microbial population [51]. In the current times, the blending of PS with hydrolysable components like starch is receiving increased attention. In the current study, we also tried to make a blend of PS and starch followed by biodegradation by our isolated fungal strain, and very promising results were found [68]. The Fourier transform infrared spectroscopy (FTIR) analysis of the PS–starch blended films confirmed the appearance of new peaks at 1223 cm^−1^, 1381 cm^−1^, and 1751 cm^−1^, which represents the C=O stretch of carbonyl groups such as ketones, aldehydes, and a carboxylic acid, and 1223 cm^−1^ to 1381 cm^−1^ represents the C-O vibrations of ester groups (Figure 7 Above).

High-pressure liquid chromatography (HPLC) analysis was applied to further quantify the biodegradation products. The presence of 1-phenyl-1,2-ethanediol and 2-phenylethanol was ascertained in all samples inoculated with fungal strain NA3 and PS–starch blend films compared to the abiotic control. The maximum concentration of 1-phenyl-1,2-ethanediol (17.7 ppm) and 2-phenyl ethanol (4.5 ppm) in the sample was obtained after 4 weeks of inoculation with *Phanerochaete chrysosporium* strain NA3 (Figure 7). At the same time, phenylacetaldehyde was found only in the fourth week of incubation of the test flask.

The literature reports that polystyrene degradation was evaluated for detection in the media [69,70,71,72]. It was apparent that 1-phenyl-1,2-ethanediol was the dominant product found in the media of the biodegradation experiments of polystyrene incubated with *Phanerochaete chrysosporium* NA3 [73] followed by 2-phenyl ethanol. When additional peaks between 1670 cm^−1^ and 1870 cm^−1^ arose, it validated the presence of previously undetected degradation products with the same functional groups as those in the control, which were also observed using FTIR. This also revealed that the biodegradation of polystyrene produced low-molecular-weight biodegradation products such as carboxylic acid, alcohols, aldehydes, and ketones that were swiftly absorbed in the medium by the fungal strain; therefore, it did not show up in the HPLC spectra or NMR spectra.

## 5. Conclusions

Management of plastic waste is a global concern, and PS is one of the leading polymers in the market. Due to the hard-to-degrade nature of the material, waste produced from this polymer is still not managed properly. In the current study, we reported the isolation and biodegradation potential of fungal strain *Phanerochaete chrysosporium* NA3. The isolated fungal strain demonstrated encouraging potential regarding the biodegradation of PS film at shake flask conditions as well as in the soil burial experiments where we bioaugmented the isolated fungus mimicking real environmental conditions. In conclusion, the current study demonstrated the possibility of the application of the newly isolated fungal strain *Phanerochaete chrysosporium* NA3 for developing a sustainable process for managing PS waste and its possible upcycling into value-added products under the concept of circular economy. Our findings indicate that it is important to identify new potential microbes that can serve as key factors regarding the development of sustainable biotechnological processes utilized for managing plastic waste.

## Figures and Tables

**Table 1:** *Phanerochaete chrysosporium* NA3.

Fungal Isolate	CO_2_ Produced in Test (g/L)	CO_2_ Produced in Control (g/L)	CO_2_ Evolved Due to Biodegradation (g/L)
***Phanerochaete chrysosporium***	19.81 ± 0.94	10.16 ± 0.63	9.65 ± 0.35

**Table 2:** Gel permeation chromatography (GPC) to evaluate the biodegradation of pure polystyrene by a fungal isolate to an abiotic control.

Treatment	Weight Average Molecular Weight Mw (Daltons)	Number Average Molecular Weight Mn (Daltons)	Polydispersity (Mw/Mn)
Control	198,066 ± 10.20	107,553 ± 10.61	1.842 ± 0.12
*Phanerochaete chrysosporium* NA3	158,169 ± 7.65	71,668 ± 9.82	2.248 ± 0.14

**Table 3:** Gel permeation chromatography (GPC) of heat-pretreated PS films compared to abiotic control.

**Heat-Pretreated Polystyrene**			
**Samples**	**Weight Average Molecular Weight**	**Number Average Molecular Weight**	**Polydispersity**
	**Mw (Daltons)**	**Mn (Daltons)**	**(Mw/Mn)**
**With no fungus (control)**	232,142 ± 9.61	73,191 ± 10.19	2.013 ± 0.09
**With *P. chrysosporium* NA3**	218,921 ± 7.53	63,909 ± 4.66	3.426 ± 0.04
**UV-Pretreated Polystyrene**			
**Samples**	**Weight Average Molecular Weight**	**Number Average Molecular Weight**	**Polydispersity**
	**Mw (Daltons)**	**Mn (Daltons)**	**(Mw/Mn)**
**With no fungus (control)**	226,780 ± 11.25	80,186 ± 10.69	2.828 ± 0.02
**With *P. chrysosporium* NA3**	203,818 ± 8.38	60,118 ± 4.08	3.39 ± 0.05

**Table 4:** Gel permeation chromatography to assess any potential reduction in the polymer’s molecular mass.

**Unsterilized Soil Burial**			
**Treatment**	**Weight Average Molecular Weight**	**Number Average Molecular Weight**	**Polydispersity**
	**Mw (Daltons)**	**Mn (Daltons)**	**(Mw/Mn)**
Unsterilized soil uninoculated control	186,069 ± 10.11	77,107 ± 5.93	2.413 ± 0.07
*P. chrysosporium* NA3	243,086 ± 8.76	72,318 ± 7.32	3.361 ± 0.08
**Sterilized Soil Burial**			
**Treatment**	**Weight Average Molecular Weight**	**Number Average Molecular Weight**	**Polydispersity**
	**Mw (Daltons)**	**Mn (Daltons)**	**(Mw/Mn)**
Sterilized soil uninoculated control	193,829 ± 5.64	94,958 ± 8.55	2.041 ± 0.08
*P. chrysosporium* NA3	189,834 ± 9.77	39,147 ± 6.71	4.849 ± 0.11

## Data Availability

The data are available within the manuscript. Further inquiries can be directed to the corresponding authors.
